# The clinicopathological significance of angiogenesis in hindgut neuroendocrine tumors obtained via an endoscopic procedure

**DOI:** 10.1186/s13000-016-0580-5

**Published:** 2016-11-08

**Authors:** Yoichiro Okubo, Osamu Motohashi, Norisuke Nakayama, Ken Nishimura, Rika Kasajima, Yohei Miyagi, Manabu Shiozawa, Emi Yoshioka, Masaki Suzuki, Kota Washimi, Kae Kawachi, Madoka Nito, Yoichi Kameda, Tomoyuki Yokose

**Affiliations:** 1 Department of Pathology, Kanagawa Cancer Center, 2-3-2, Nakao, Asahi-Ku, Yokohama, Kanagawa 241-8515 Japan; 2 Department of Gastroenterology, Kanagawa Cancer Center, 2-3-2, Nakao, Asahi-Ku, Yokohama, Kanagawa 241-8515 Japan; 3 Molecular Pathology and Genetics Division, Kanagawa Cancer Center Research Institute, 2-3-2, Nakao, Asahi-Ku, Yokohama, Kanagawa 241-8515 Japan; 4 Department of Gastrointestinal Surgery, Kanagawa Cancer Center, 2-3-2, Nakao, Asahi-Ku, Yokohama, Kanagawa 241-8515 Japan; 5 Department of Thoracic Surgery, Kanagawa Cancer Center, 2-3-2, Nakao, Asahi-Ku, Yokohama, Kanagawa 241-8515 Japan

**Keywords:** Neuroendocrine tumor, Hindgut, Microvessel, Angiogenesis

## Abstract

**Background:**

As the World Health Organization grading system for gastroenteropancreatic-neuroendocrine tumors (GEP-NETs) may not always correlate with tumor progression, it is imperative that other independent predictors of tumor progression be established. To identify such predictors, we conducted a retrospective histopathological study of hindgut NETs, obtained from endoscopic procedures, and used statistical analyses to evaluate predictive factors.

**Methods:**

We first obtained clinicopathological data of cases of hindgut NETs. Tissue sections from tumor samples were prepared and subjected to pathological examination. In particular, we calculated the microvessel density (MVD) and lymphatic microvessel density (LMVD) values, and performed appropriate statistical analyses.

**Results:**

A total of 42 cases of hindgut NETs were selected for the study, 41 from the rectum and 1 from the sigmoid colon. Based on the Ki-67 labeling index, 34 cases were classified as NET G1 tumors and 8 as NET G2 tumors. MVD values ranged from 1.4/mm^2^ to 73.9/mm^2^ and LMVD values from 0/mm^2^ to 22.9/mm^2^. MVD and LMVD were identified as risk factors for venous and lymphatic invasion of hindgut NETs. Moreover, MVD positively correlated with the maximum diameter of the tumor.

**Conclusions:**

Tumor progression of NETs may cause angiogenesis and lymphangiogenesis, via an unknown mechanism, as well as lymphovascular invasion. Angiogenesis likely plays an important role in occurrence and progression in the initial phase of hindgut NETs.

## Background

Although previous investigators have reported on the prognostic value of lymphovascular invasion in several types of neoplasms, the results of these studies are somewhat controversial with regards to gastroenteropancreatic neuroendocrine tumors (GEP-NET) [1–3]. However, a number of recent studies have reported a higher than expected rate of venous and/or lymphatic invasion (lymphovascular invasion) in GEP-NET samples obtained via endoscopic procedures [4–6]. In a case series from our own institution, we identified a lymphovascular invasion rate of approximately 30 % for GEP-NETs. Therefore, we conducted this study to specifically evaluate the lymphovascular status of GEP-NETs.

GEP-NETs have traditionally been regarded as relatively rare neoplasms [7]. However, recent surveillance data have shown the incidence and prevalence of GEP-NETs to be higher than previously expected, likely due to recent technical advances in endoscopic and imaging examinations [8]. In Japan, the rectum is the most frequent site of GEP-NETs [8], as well as the second most common site in Western countries [4].

The grading system for GEP-NETs was updated by the World Health Organization (WHO) in 2010 [9–11]. This system is based only on the proliferative activity of tumor cells, as measured by the number of mitotic cells confirmed per 10 high-power fields (HPFs) and/or by the percentage of tumor cells showing positive immunoreactivity for the Ki-67 antigen (the Ki-67 labeling index). However, the proliferative characteristics of tumor cells may not always predict tumor progression or of its growth, invasion and metastasis and overall prognosis [12]. Therefore, it is imperative that other independent predictors of tumor progression be established.

In Japan, NETs mostly originate in the hindgut, with a relatively well-established endoscopic procedure used in most of these cases to remove these hindgut NETs [4, 13]. Previous study [8] has reported an overall prevalence of GI-NETs of 6.42 per 100,000 people (95 % confidence interval (CI), 4.50 to 8.34). The specific prevalence rates of foregut, midgut, and hindgut NETs were estimated at 1.67 (95 % CI, 0.94–2.40), 0.23 (95 % CI, 0.18–0.28), and 4.52 (95 %, CI 3.17–5.87) per 100,000 people, respectively. In Japan, hindgut NETs account for approximately 70 % (70.4 %) of all GI-NETs (Table 1). Therefore, the aim of our retrospective study was to perform histopathological and statistical analyses of hindgut NETs, obtained by endoscopic procedures, to identify independent predictors of tumor progression and prognosis, based on pathological findings.

**Table 1:** Epidemiology of gastrointestinal neuroendocrine tumors in Japan (per 1000,000 population)

2010 (JAPAN)	
Overall prevalence of GI-NETs	6.42 (95 % CI 4.50–8.34)
Foregut	1.67 (95 % CI 0.94–2.40)
Midgut	0.23 (95 % CI 0.18–0.28)
Hindgut	4.52 (95 % CI 3.17–5.87)
Incidence of GI-NETs	3.51 (95 % CI 2.50–4.53)
Foregut	1.20 (95 % CI 0.48–1.91)
Midgut	0.15 (95 % CI 0.12–0.18)
Hindgut	2.12 (95 % CI 1.56–2.67)

Legend: The foregut included the esophagus, stomach and duodenum; the midgut, the jejunum, ileum and vermiform appendix; and the hindgut, the large intestine and colon

GI-NET, gastrointestinal-neuroendocrine tumor; 95 % CI, 95 % confidence interval

## Methods

### Identification of hindgut NETs cases for analysis

We searched for cases of hindgut NETs, recorded between April 1996 and September 2015, using a pathological diagnosis support software (‘EXpath’ System, INTEC Inc., Tokyo, Japan). The following keywords were used for the search: ‘carcinoid’, ‘neuroendocrine’, ‘karuchinoid’ (Japanese for carcinoid), and ‘shinkeinaibunpi’ (Japanese for neuroendocrine). The terms ‘ohkokettyou, Sjoketyou and tyokutyou, and S’ (Japanese for transverse colon, sigmoid colon, and rectum) were used as an additional option to identify the tumor site. We subsequently retrieved the formalin-fixed paraffin-embedded (FFPE) tissue sections of identified hindgut NET cases.

### Clinicopathological data of identified hindgut NET cases

We extracted the following clinical data from the medical records of identified hindgut NET cases for analysis: age, sex, and outcome. We also extracted reports of pathological findings for review. To conduct the pathological assessment of retrieved specimens, tissue sections from tumors were prepared and subjected to Hematoxylin and Eosin (HE) staining for analysis under a light microscope. Immunohistochemical examinations were performed using antibodies against the following markers: CD31 (Leica, clone 1A10; 1:20 dilution), chromogranin A (Roche, clone LK2H10; 1:5 dilution), D2-40 (Roche, clone D2-40; 1:1 dilution), Ki-67 (Dako, clone MIB-1; 1:50 dilution), and synaptophysin (Roche, clone MRQ-40; 1:1 dilution). Tumors were defined as NETs if immunoreactivity staining was positive for synaptophysin and/or chromogranin A. The Ki-67 labeling index was obtained by counting at least 1000 cells in each case, using the Patholoscope image analysis software (MITANI Corporation, Japan, URL: http://www.mitani-visual.jp/en/products/bio_imaging_analysis/patholoscope/).

For analysis, microscope images were captured at the center of the tumor primarily under two magnifications. Low and High power fields. However, other magnifications (×100 or ×200) were used for certain cases to produce multi-panel figures. This was necessary due to the relatively small number of specimens available for analysis and the absence of burtons surrounding stromal tissues. A scaling bar was included on all images to explain length measurements (and these are provided in the Figure legends). We examined the following additional pathological data as surrogate measures of tumor progression: tumor site, maximum tumor diameter, depth of tumor invasion, surgical margin status, and the status of lymph node metastasis.

Based on our identification of variation in the density of vessels and lymphatic vessels during histopathological examination of our cases, we selected to specifically calculate the microvessel density (MVD) and lymphatic microvessel density (LMVD) values in tumor specimens. MVD is defined as the number of blood vessels per unit area of tumor tissue, while LMVD refers to the number of lymphatic vessels per unit area. Previous investigators have reported that MVD may be one of the prognostic factors of the NETs [14].

To obtain these vessel counts, immunohistopathological images of the tumor stained for CD31 (for MVD count) or D2-40 (for LMVD count) were captured using a video microscope camera (DS-Fi1, Nikon, Tokyo, Japan), and the count carried out manually. CD31- and D2-40-positive structures were defined as vessels if a luminal structure was identified; single cells, positive for CD31 or D2-40, were not included. MVD and LMVD were counted in 20 HPFs of histopathological images, using the hot spot counting method. Counting was facilitated by the specificity of CD31 for vascular endothelial cells, which is higher than when using CD34. For LMVD, although we recognized the low specific of D2-40 for lymphatic vessels, we used this staining technique because of its simplicity and instrumentation, as recognized by the Japanese Society for Cancer of the Colon and Rectum (URL:http://www.kanehara-shuppan.co.jp/books/detail.html?isbn=9784307203142; in Japanese).

### Statistical analyses

Appropriate statistical analyses were performed on the extracted data. Statistical analyses were performed using the non-parametric Mann-Whitney *U* test or Pearson’s product-moment correlation coefficient, as appropriate for the data set. Differences were considered significant at *P* < 0.05.

## Results

### Hindgut NETs cases included in the analysis

Forty-seven relevant hindgut NET cases were identified, with specimens available for retrieval and analysis. Of these 47 specimens, a tumor lesion was identified in 42 of the collected FFPE tissue sections, with insufficient tumor tissue available for examination in the remaining 5 cases. The analysis was, therefore, based on the histopathological data of 42 cases of hindgut NETs.

### Clinicopathological data of hindgut NET cases

**Fig. 1:**
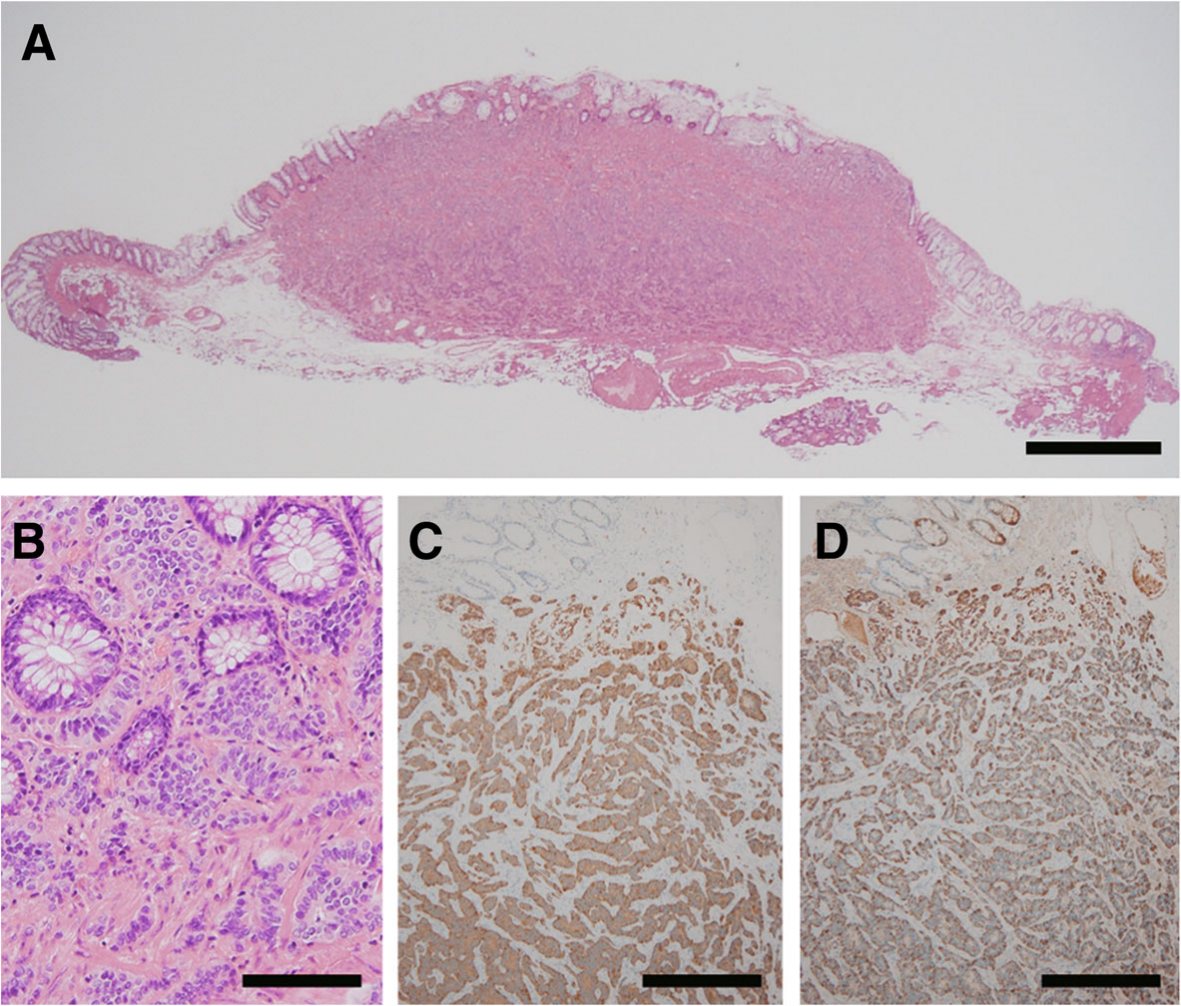
Representative histopathological findings of hindgut neuroendocrine tumors. Legend: **a** A photomicrograph showing a low-power field of a hindgut neuroendocrine tumor (NET), with evidence of invasion of the submucosal layer (hematoxylin and eosin (HE) staining; original magnification, ×20; scale bar represents 1000 μm). **b** A photomicrograph showing a high-power field of a hindgut NET. The tumor cells have a round-to-oval nucleus, and the nuclear atypia is relatively mild (HE staining; original magnification, ×400; scale bar represents 100 μm). **c** and **d** Among the 42 cases in our study, a positive immunoreactivity for synaptophysin was identified in 42 cases and for chromogranin A in 35 cases (immunohistochemistry, synaptophysin and chromogranin A; original magnification, ×100; scale bars represent 200 μm)

**Fig. 2:**
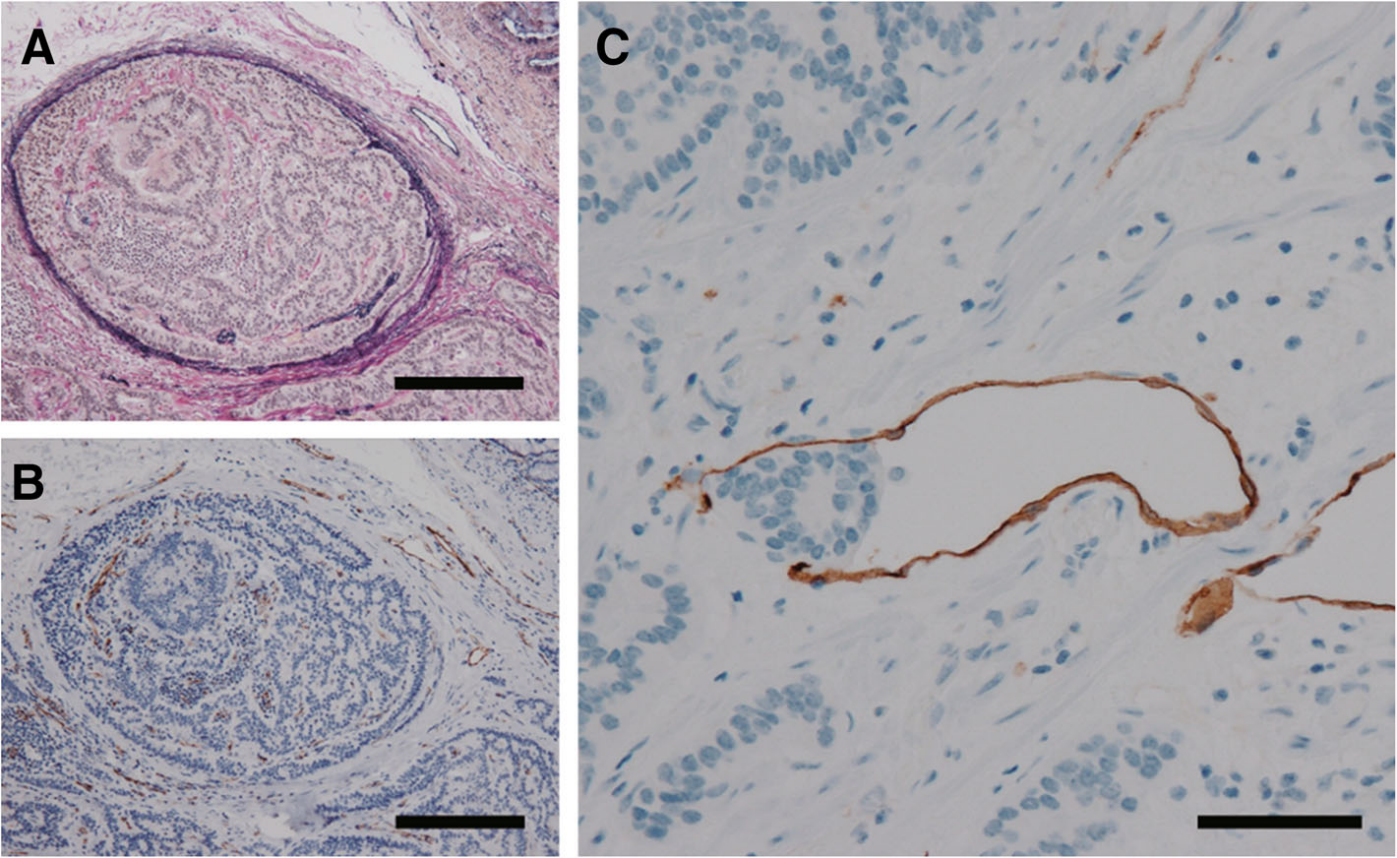
Representative images of venous and lymphatic invasion. Legend: **a**, **b** To determine the presence or absence of venous invasion, both EVG staining and immunohistochemistry for CD31 were performed. Venous invasion was confirmed, but no CD31-positive cells were identified. Such difficult cases were reviewed by more than two expert pathologists (EVG staining and immunohistochemistry for CD31; original magnification, ×200; scale bars represent 200 μm). **c** To determine the presence or absence of lymphatic invasion, immunohistochemistry for D2-40 was performed. Difficult cases were examined by more than two expert pathologists (Immunohistochemistry for D2-40; original magnification × 400; scale bar represents 100 μm)

**Fig. 3:**
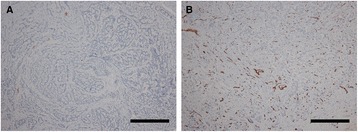
Variation in microvessel density among tumors. Legend: **a** In this tumor, there are few CD31-positive endothelial cells and the tumor has an extremely low microvessel density. No venous invasion was observable (immunohistochemistry for CD31; original magnification, ×100, scale; bar represents 100 μm). **b** In this tumor, numerous CD31-positive endothelial cells were observable and the tumor has a high microvessel density. This is representative of most of cases with venous invasion (immunohistochemistry for CD31; original magnification, ×100; scale bar represents 100 μm)

**Fig. 4:**
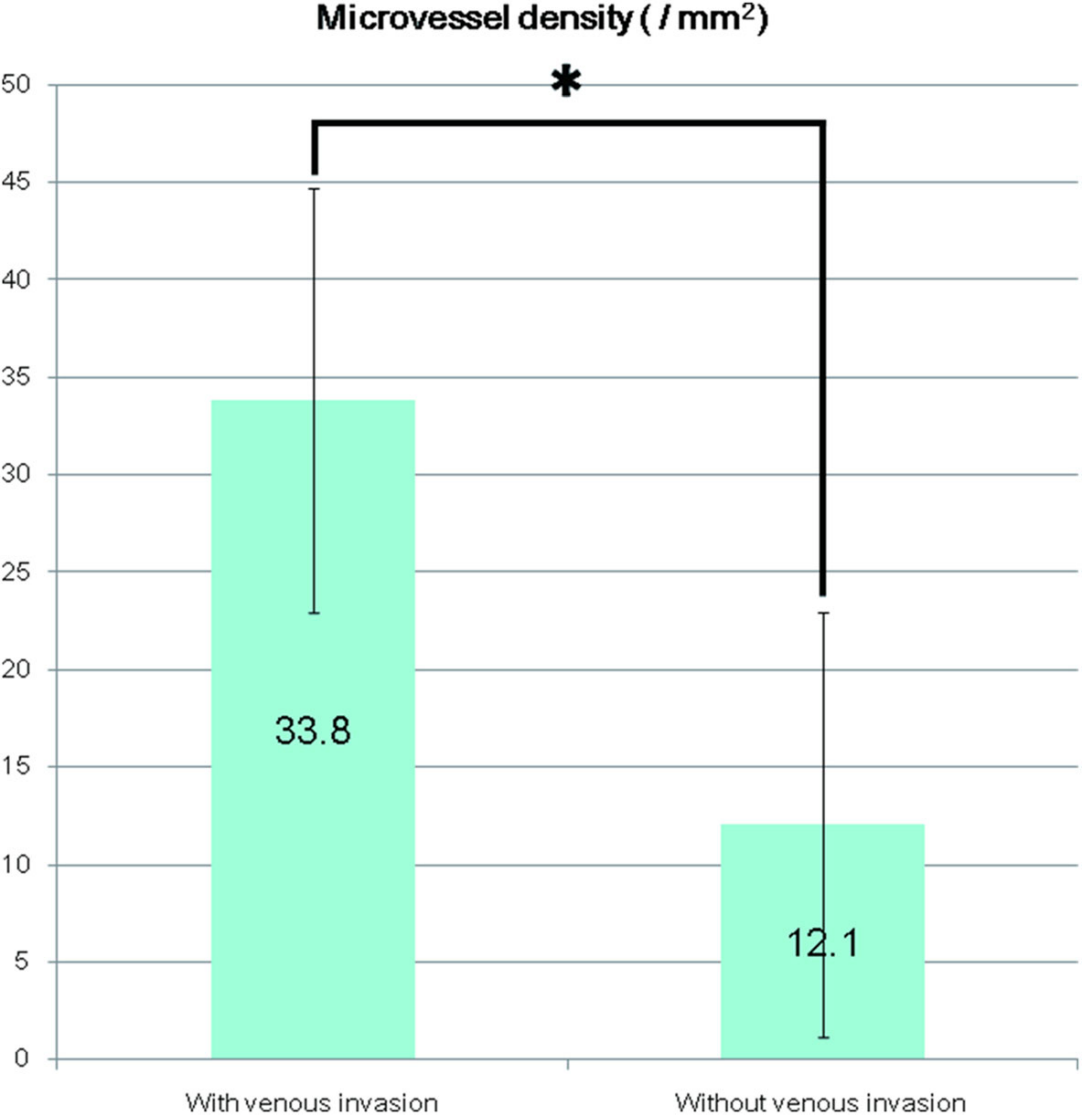
Differences in microvessel density in tumors *with* and with *without* venous invasion. Legend: The microvessel density values of tumors *with* venous invasion were significantly higher than for tumors *without* venous invasion (*, *p* < 0.001, Mann–Whitney *U* test; values are expressed as the mean ± standard deviation)

**Fig. 5:**
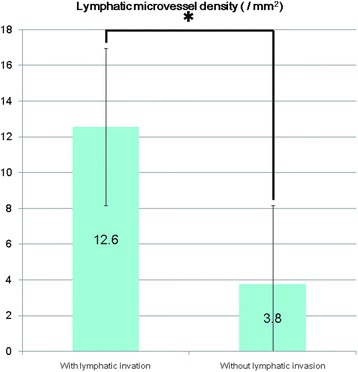
Differences in lymphatic microvessel density in tumors *with* and *without* lymphatic invasion. Legend: The lymphatic microvessel density values of tumors *with* lymphatic invasion were significantly higher than for tumors *without* lymphatic invasion (*, *p* < 0.001, Mann–Whitney *U* test; values are expressed as the mean ± standard deviation)

The relevant clinical data were as follows: a mean ± standard deviation (SD) of age of 59.6 ± 12.0 years (range, 37 to 81 years) and a male-to-female ratio of 23:19. No deaths from hindgut NETs were reported over the follow-up period, which ranged between 5 and 191 months. Four patients with hindgut NET underwent further surgical intervention due to lymphatic invasion detected in the specimen obtained during the initial endoscopic procedure. In 1 of these 4 patients, lymph node metastasis occurred in a level 1 lymph node. A positive surgical margin (vertical margin positive) was identified in another case, the patient refusing additional surgical intervention, but rather opting for observation and careful follow-up. This patient maintained a high quality of life over his 36 months of follow-up, after the endoscopic procedure, with no evidence of recurrent local or distant metastases. Four patients died of other disease causes, 2 of gastric cancer, 1 of lung cancer and 1 of cerebral hemorrhage. Overall, among the 42 cases of NETs, clinical characteristics were available in the medical record for only 22 patients who underwent regular medical follow-ups after the endoscopic procedure. With regards to pathological findings, 41 of the 42 cases of hindgut NETs occurred in the rectum and 1 in the sigmoid colon. The maximum diameter of the tumors ranged between 998.1 and 10046.0 μm (mean ± SD, 5058.0 ± 2410.3 μm). The depth of tumor invasion was the submucosal layer in all 42 cases (Figs. 1a and b). On immunohistochemical examination, positive immunoreactivity for synaptophysin was identified in all 42 cases (100 %) and for chromogranin A was in 35 of 42 (83.3 %) cases (Fig. 1c and d). The Ki-67 labeling index ranged between 0.1 and 3.2 % (mean ± SD, 1.1 ± 0.8 %). On the basis of the Ki-67 labeling index, 34 cases were classified as NET G1 tumors and 8 as NET G2 tumors. Venous invasion was identified in 10 of 42 cases (23.8 %) and lymphatic invasion in 13 of 42 cases (31.0 %; Fig. 2). MVD and LMVD values varied among tumors (Fig. 3). MVD ranged between 1.4/mm^2^ and 73.9/mm^2^ (mean ± SD, 17.3 ± 14.2/mm^2^, Fig. 4) and LMVD from 0/mm^2^ to 22.9/mm^2^ (mean ± SD, 6.5 ± 6.5/mm^2^, Fig. 5). These data are summarized as Table 1.

### Statistical analyses findings

To identify independent predictors of tumor progression, such as invasion and/or metastasis in hindgut NETs, we evaluated the predictive valued of MVD and LMVD. MVD values were higher in tumors with venous invasion (mean ± SD, 33.8/mm^2^ ± 20.7/mm^2^ than in tumors without venous invasion (12.1/mm^2^ ± 5.3/mm^2^; *p* < 0.001, Mann-Whitney *U* test; Fig. 4). Moreover, a positive correlation was identified between MVD and the maximum diameter of the tumor (*r* = 0.612, *p* < 0.001, Pearson’s product-moment correlation coefficient; Table 2). Additionally, LMVD values were higher in tumors with lymphatic invasion than in tumors without lymphatic invasion (12.6/mm^2^ ± 6.9/mm^2^ versus 3.8/mm^2^ ± 4.1/mm^2)^; *p* < 0.001, Mann-Whitney *U* test; Fig. 5). No significant correlation was identified between LMVD and the Ki-67 labeling index values or the maximum tumor diameter (*p* = 0.744 and *p* = 0.136, respectively, Pearson’s product-moment correlation coefficient; Table 2). Due to the small number of cases, only univariate analyses of MVD and LMVD could be performed, and effects of age, grading, and sex could not be specifically evaluated.

**Table 2:** Clinicopathological chracteristics of hindgut neuroendocrine tumor

Age (years)	
Range	37 to 81
mean ± SD	59.6 ± 12.0
MVD (mm^2^)	
Range	1.4 to 73.9
mean ± SD	17.3 ± 14.2
Relationship to venous invasion	Significant positive correlation (Mann–Whitney *U* test)
LMVD (mm^2^)	
Range	0 to 22.9
mean ± SD	6.5 ± 6.5
Relationship to lymphatic invasion	Significant positive correlation (Mann–Whitney *U* test)
Gender (n, %)	
Male	23, 54.8 %
Female	19, 45.2 %
Pathological grade (n, %)	
NET G1	34, 81 %
NET G2	8, 19 %
Ki 67 labeling index (%)	
Range	0.1 to 3.2
mean ± SD	1.1 ± 0.8
Relationship to tumor diameter	No significant correlation (Pearson’s product–moment correlation coefficient, *p* = 0.136)
Venous invasion (n, %)	
Negative	32, 76.2 %
Positive	10, 23.8 %
Lymphatic invasion (n, %)	
Negative	29, 69 %
Positive	13, 31 %
Tumor diameter (μm)	
Range	998.1 to 10046.0
mean ± SD	5058.0 ± 2410.3
Relationship to MVD	Significant positive correlation (Pearson’s product–moment correlation coefficient, *r* = 0.612, *p* < 0.001)
Relationship to LMVD	No significant correlation (Pearson’s product–moment correlation coefficient, *p* = 0.744)
Pathological stage (n, %)	
pStage I	40, 95.2 % (all of them are pT1 and n0)
pStage II	0, 0 %
pStage IIIB	1, 2,4 % (lymph node metasis positive)
Unknown	1, 2.4 % (margin positive)

Legend: In this table clinicopathological characteristics and some statistical analyses of 42 hindgut neuroendocrine tumors were summarized

*MVD* microvessel density, *LMVD* lymphatic microvessel density, *SD* standard deviation

## Discussion

In this study, we specifically calculated the MVD and LMVD to confirm the presence or absence of lymphovascular invasion in hindgut NET cases, and performed statistical analyses to examine the relationship between these values and the Ki-67 labeling index and the maximum tumor diameter. Morphometric analysis further revealed MVD and LMVD to be risk factors of venous and lymphatic invasion in the initial phase of hindgut NETs. This latter finding results is explained by the nature of our samples, with the maximum tumor diameter being <10 mm in most cases.

Our findings provide evidence on the relationship between tumor progression and angiogenesis. Although it is widely accepted that the Ki-67 labeling index generally reflects tumor progression [15, 16], we did not identify a significant correlation between the Ki-67 labeling index and the maximum tumor diameter. In contrast, we did identify a significant positive correlation between MVD and maximum tumor diameter, indicative of possible common mechanisms underlying NET progression and angiogenesis in GEP-NETs. In addition, as venous invasion of NETs is a risk factor of metastasis, this mechanism could influence not only the progression of NETs but also the spread of distant metastases (especially liver metastasis). Our analyses revealed a significant positive correlation between the maximum tumor diameter and MVD. Previous investigators have also reported that MVD may be one of the prognostic factors of NETs [14]. The plausible effect of angiogenesis on tumor progression is further supported by absence of a correlation between the LMVD and Ki-67 labeling index values or the maximum tumor diameter.

Based on this evidence of a plausible effect of angiogenesis on tumor progression, novel angiogenesis targeting agents could prove beneficial in the treatment of NETs. Future research should include molecular, biological, and genetic analyses, such as the angiogenesis-related genes, to more comprehensively identify novel independent factors of tumor progression, as well as to inform the development of new, and likely more effective, treatment strategies.

The limitations of our study need to be considered in the interpretation of our results. Foremost, this is a retrospective case series and, therefore, is subject to the inherent biases of this research design. Moreover, our analysis was based on only 42 cases of NETs, which prohibited the use of multivariate analyses to further explore the predictive effects of age, sex, and tumor staging, among other relevant clinicopathological characteristics.

Despite these limitations, our study provides evidence of a plausible role of angiogenesis in the occurrence and progression in the initial phase of hindgut NETs. Our findings provide a basis for future studies examining the role of angiogenesis-related genes and of targeted gene therapies as novel treatments for NETs.

## Conclusions

Our study indicated that angiogenesis mechanism play important roles in occurrence and progression in the initial phase of hindgut NETs. Furthermore, our data indicated that molecular, biological, and genetic analyses, such as the examination of angiogenesis-related genes, might provide efficient and new research strategies to elucidate the progression of NETs as well as identify novel independent predictors of these tumors.

## Abbreviations

GEP-NET: Gastroenteropancreatic neuroendocrine tumor
WHO: World Health Organization
HPF: High-power field
FFPE: Formalin-fixed paraffin-embedded
HE: Hematoxylin and Eosin
MVD: Microvessel density
LMVD: Lymphatic microvessel density
SD: Standard deviation
